# Potential Molecular Mechanisms of Alcohol Use Disorder with Non-Coding RNAs and Gut Microbiota for the Development of Superior Therapeutic Application

**DOI:** 10.3390/genes15040431

**Published:** 2024-03-29

**Authors:** Moeka Nakashima, Naoko Suga, Sayuri Yoshikawa, Yuka Ikeda, Satoru Matsuda

**Affiliations:** Department of Food Science and Nutrition, Nara Women’s University, Kita-Uoya Nishimachi, Nara 630-8506, Japan

**Keywords:** ncRNA, lncRNA, miRNA, autophagy, gut microbiota, gut-brain axis, alcohol dependence, alcohol use disorder

## Abstract

Many investigations have evaluated the expression of noncoding RNAs (ncRNAs) as well as their related molecular functions and biological machineries in individuals with alcohol dependence. Alcohol dependence may be one of the most prevailing psychological disorders globally, and its pathogenesis is intricate and inadequately comprehended. There is substantial evidence indicating significant links between multiple genetic factors and the development of alcohol dependence. In particular, the critical roles of ncRNAs have been emphasized in the pathology of mental illnesses, probably including alcohol dependence. In the comprehension of the action of ncRNAs and their machineries of modification, furthermore, they have emerged as therapeutic targets for a variety of psychiatric illnesses, including alcohol dependence. It is worth mentioning that the dysregulated expression of ncRNAs has been regularly detected in individuals with alcohol dependence. An in-depth knowledge of the roles of ncRNAs and m6A modification may be valuable for the development of a novel treatment against alcohol dependence. In general, a more profound understanding of the practical roles of ncRNAs might make important contributions to the precise diagnosis and/or actual management of alcohol dependence. Here, in this review, we mostly focused on up-to-date knowledge regarding alterations and/or modifications in the expression of ncRNAs in individuals with alcohol dependence. Then, we present prospects for future research and therapeutic applications with a novel concept of the engram system.

## 1. Introduction

Alcohol dependence, which may include alcohol use disorder and/or alcohol abuse, is a kind of neuropsychiatric disease which can be described in terms of being unable to stop drinking without suffering withdrawal symptoms and/or continuing alcohol use in spite of destructive consequences. The representative symptoms may include habitual alcohol use, loss of control over drinking, and alcohol withdrawal symptoms [1,2]. Genes may be responsible for around half of the risk of alcohol dependence [1]. The consequences are connected with extensive disability as well as substantial medical and/or economic burdens [3]; for these reasons, alcohol dependence is one of the most widespread mental illnesses globally [4]. Hereafter, the term alcohol dependence is practically equated with alcohol addiction, alcohol abuse, alcoholism, and/or alcohol use disorder.

**Figure 1:**
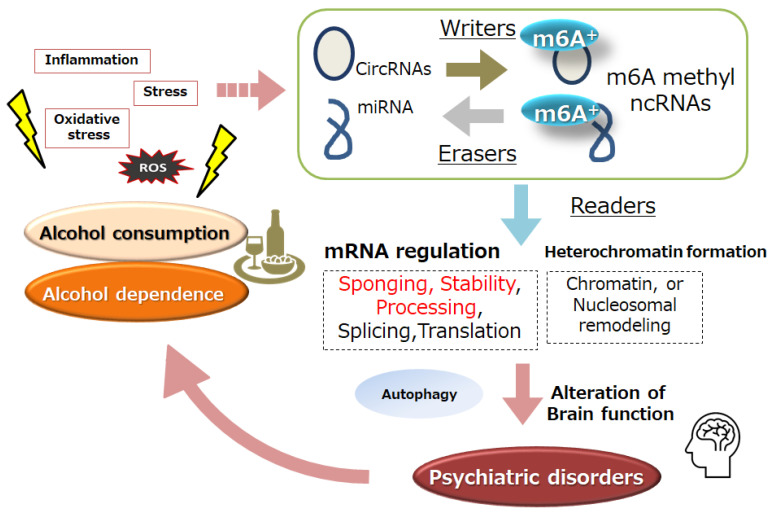
Representation of the association of non-coding RNAs (ncRNAs) and m6A modification (m6A^+^) of ncRNAs with psychiatric disorders and/or alcohol dependence. In the beginning, the m6A modification may be controlled by methyltransferases “writers” and demethylases “erasers” by the stimulation of inflammation and/or oxidative stress with reactive oxygen species (ROS). The ncRNAs and m6A-ncRNAs with binding “readers” molecules may contribute to several activities of RNAs, including sponging, stability, processing, and/or translation of mRNAs, which could be consequently an important process in several psychiatric disorders, including alcohol dependence. Note that several important activities of m6A, such as heterochromatin formation via the regulation of histone methyltransferase, have been partially omitted for clarity.

Chronic alcohol exposure produces general neuroadaptations or alterations in gene expression in individuals [5]. It has been hypothesized that non-coding RNAs (ncRNAs) may participate in the regulatory network that affects potential molecular targets of certain signaling pathways that control biological and cellular outcomes, eventually leading to the incidence and/or progress of alcohol dependence. ncRNAs are bioactive molecules in organisms that mediate multiple biological processes including mRNA splicing, the regulation of translation, and/or post-transcriptional modification for altered intracellular signal transduction [6]. Biotechnological investigations have shown that ncRNAs are typically abundant in the central nervous system (CNS) and play a key role in emotional homeostasis as well as the pathological processes of psychiatric diseases via epigenetic mechanisms [7]. ncRNAs have been shown to regulate a variety of ion channels and/or intercellular connecting proteins/molecules. Many distinctive sequences of ncRNA may occur within a cell, which can form a segment of the background of transcriptional machineries [8]. ncRNAs are the well-designed regulatory gears of gene expression with mixed some groups, which could be additionally divided into microRNAs (miRNAs), circular RNAs (circRNAs), long non-coding RNAs (lncRNAs), small interfering RNAs (siRNAs), piwi interacting RNAs (piRNAs), and so forth [9]. Investigations into the possible roles of these ncRNAs in the progress of several brain disorders have made, positioning ncRNAs as potential tools for innovative therapeutic approaches. The dysregulation of circRNAs, miRNAs, and/or lncRNAs has been detected in human patients with certain psychological disorders, which might be associated with the inception and/or progress of various psychiatric illnesses, including alcohol dependence [10] (Figure 1).

Epigenetics is a field that studies genetic changes in gene expression that do not include changing the DNA sequence. The foremost epigenetic mechanisms contain the well-known regulation by above-mentioned ncRNAs, histone modifications, and DNA/RNA methylation; the understanding of these epigenetic mechanisms is an area of continuing investigation. In particular, a diverse range of studies have suggested that ncRNAs may play an important role in epigenetic control [11]. In addition to genetic variation, various stressors including psychological stressors and environmental social factors can lead to alcohol dependence via epigenetic modifications at the transcriptional level of RNAs [12,13,14,15]. Herein, we summarize recent research developments with the purpose of coming to a better understanding of ncRNAs/epigenetics and their mechanisms during the pathogenesis of alcohol dependence, which may contribute to gaining a comprehension of the underlying mechanisms of alcohol dependence. In turn, this will support the development of improved tactics against alcohol dependence.

## 2. Alcohol Dependence and ncRNA

The expression analysis/profile of miRNAs has shown that some miRNAs are abnormally expressed in patients with psychological disorders, which may also be involved in the progression of alcohol dependence through several biological mechanisms. For example, more than 35 miRNAs including hsa-miR-553 and let-7f are considerably upregulated in individuals with alcohol dependence compared with healthy controls [16]. Plasma miRNA profiling/analysis has also shown that the plasma concentrations of miR-193b-3p, miR-122-5p, miR-3937, and miR-4507 are associated with alcohol intake, which might play a key role in the pathogenesis of alcohol dependence [17]. The miRNA-dependent regulation of target genes might be also critical for the pathogenesis of alcohol dependence.

Likewise, dysregulated lncRNAs may be found in the brain of individuals with alcohol dependence; this dysregulation may contribute to the abnormal production of brain-derived neurotrophic growth factor (BDNF) in individuals with alcohol dependence [18]. The expression of several lncRNAs including SNORD3C, HSPA7, and RP11-543H23.2 has been aberrantly detected in different brain regions of patients with alcohol dependence. Some lncRNAs including NCRNA-00051 or 00176 and 00107 are also more highly expressed in the prefrontal cortex of individuals with alcohol dependence compared with healthy controls. These lncRNAs may be related to the dysfunction of splicing factors by regulating some post-transcriptional processes [19]. There are more than a few lncRNAs which are significantly decreased in individuals with alcohol dependence [20].

Furthermore, an accumulating quantity of research has also emphasized the view that circRNAs may be diagnostic markers for alcohol dependence. The expression levels of serum hsa-circ-0004771 in patients with alcohol dependence are considerably higher than those in normal controls. Therefore, hsa-circ-0004771 could be a diagnostic biomarker. Differently expressed circRNAs may interact with various alcohol dependence-related miRNAs, which could influence several inflammatory pathways [21]. For example, miR-1200 and/or circRNA-406742 can meaningfully interact with various mRNAs associated with neuronal functioning, psychiatric disorders, and/or alcohol addiction [21,22]. Interestingly, the decreased expression of circRNA-406742 can be found in patients with alcohol dependence, which is negatively correlated with the expression of miR-1200 [21]. CircRNAs may function as a sponge of miRNAs to affect the function of neurons through the regulation of several target genes.

In these ways, a large body of research, which was conducted using biotechnological approaches, has emphasized the critical roles of several ncRNAs in the pathophysiology of mental illnesses and alcohol dependence [7,23]. About 10% of miRNAs are downregulated in alcoholism, including miR-126, miR-153, miR-432, and miR-567, suggesting that GABA and/or dopamine-related miRNAs are upregulated in alcoholism [23]. Unlike the relatively stable genetic code, this combinatorial ncRNAs’ epigenetic code may be vigorously reprogrammed as a cause or consequence of psychiatric disorders and/or alcohol dependence [24]. From normal development and physiology to the regulation of diseases including alcoholism and/or several psychiatric disorders, some ncRNA molecules have been discovered to mediate diverse processes in the CNS [5,25]. For example, ncRNAs may be developed as therapeutic agents to protect the blood–brain barrier of patients with CNS damage [25].

## 3. Alcohol Dependence and m6A Modification of RNAs

Alterations in the epitranscriptome could authenticate its investigation as an imperative modulator. The epitranscriptome encompasses all post-transcriptional modifications that occur on RNAs. The most prevalent modification is the methylation of N6 adenosine (m6A) that occurs on specific sequence contexts of RNAs [26], which can change the function and/or regulation of their RNA targets. Environmental factors including anxiety, stress, and/or social pressure could lead to alcohol dependence through chromatin remodeling and/or epigenetic regulation. Some of these environmental factors could also lead to epigenetic modifications within post-transcriptional levels. The m6A modification is the most well-known modification of RNAs, which could control transcript stability, splicing, translation, and the association with ncRNAs. [27]. Continuing ethanol exposure may alter the methylation levels of RNAs, mRNA methylation, and/or expression levels of mRNAs, suggesting a potential epitranscriptomic mechanism by which continuing alcohol consumption may remodel the expression of interrelated alcohol-responsive genes, thus enhancing the risk of developing alcohol dependence [28]. Strikingly, in the postmortem amygdala of patients with early onset alcohol dependence, brain-derived neurotrophic factor (BDNF)*-antisense* lncRNA is hypomethylated, leading to decreased expression levels of BNDF [29]. In the context of alcohol intoxication, the upregulation of the lncRNA could ameliorate BDNF expression, in which BDNF-AS seems to be regulated by diminished levels of m6A [29]. BDNF is a member of the neurotrophin family, with well-known roles in neural development and synaptic plasticity. Therefore, lncRNAs play significant roles in the regulation of BDNF expression [30].

The m6A modification is a ubiquitous mRNA modification in eukaryotes, which occurs through the action of methyltransferases, demethylases, and methylation-binding proteins. The m6A methylation of RNAs is associated with various neurological disorders including depression, epilepsy, Parkinson’s disease, Alzheimer’s disease, brain injury, and brain gliomas. In addition, it has been shown that RNA m6A modification may play key roles for regulating chromatin states and gene expression, which might be involved in many important biological processes in health and disease [31]. Therefore, m6A-related drugs have received increasing attention in the therapeutic treatment of neurological disorders. A number of signaling pathways in brain have been found to be mediated through m6A, but only a few studies have directly investigated the effects of m6A on depression and/or depressive-like behaviors. Depression is a common psychiatric disorder described by continued low mood, which may be associated with m6A methylation. Therefore, the m6A-related molecules including METTL3, METTL14, ALKBH5, and WTAP are associated with major depression [32]. In addition, the gene expression level of m6A controllers has been associated with depressive-like behaviors [33]. Interestingly, it has been reported that the regulation of m6A is compromised in major depressive disorder patients following glucocorticoid receptor stimulation [27,34].

Related proteins of m6A modification could play key roles in the development of various neuropsychiatric disorders including depression, Parkinson’s disease, and Alzheimer’s disease. The m6A modification regulation mechanism in the CNS during the development of neuropsychiatric disorders may provide some insight into new research targets and treatment directions [35]. Similarly, the disruption of m6A modification may be one of the most important causes for the abnormal function of the CNS, leading to the occurrence of CNS disorders such as depression [36]. Drinking too much alcohol may lead to neuronal atrophy, which is associated with an increased risk for anxiety, depression, cognitive deficits, and the altered regulation of drinking behaviors [37]. In addition, chronic stress, anxiety, and depression may be key risk factors for developing alcohol dependence [38]. In fact, depression is often comorbid with alcohol dependence with severe stress components [39] (Figure 1).

## 4. Individual Epigenetic Mechanisms for Alcohol Dependency

There is a huge body of evidence showing that alcohol can alter gene expression through epigenetic processes [40]. Epigenetic mechanisms, such as the acetylation of the N-terminal tails of histones that pack up DNAs to nucleosomal remodeling, could cause transcriptional change in addiction, which may direct related genes in specific brain regions to contribute to the production of helpful phenotypes associated with alcohol tolerance. Studies that use alcohol withdrawal to induce depressive-like behaviors have adopted different routes and intervals of alcohol exposure and withdrawal. However, a relationship might exist between the individual sensitivity to the aversive properties of ethanol and the risk for alcohol dependency. An important confusing factor to deliberate about is that the molecular changes induced by alcohol consumption itself and withdrawal from habitual alcohol use may not be related to depressive-like behaviors. Therefore, it might be important to establish a causal role for specific epigenetic mechanisms and alterations of gene expression explicitly induced by alcohol in depressive-like conditions [41]. Epigenetic mechanisms may also play an imperative role in depression [42]. Analytical methods of genome-wide DNA methylation and histone modification profiles have delivered respected information to establish the functional role of histone modification on specific genes [43]. Alcohol dependency may actively lead to relaxed chromatin due to the downregulation of DNA/histone methylation. Otherwise, chronic exposure might in part lead to a close-fitting chromatin bundle. Consequently, alcohol drinking may affect epigenetic mechanisms responsible for adaptation alterations of several brain paths probably linked to stress management [44]. After withdrawal, chromatin may tend to the condensed state via the upregulation of DNA and/or histones [45]. For example, mRNA expression levels are significantly lower compared to controls, which correspond to alterations in DNA methylation in a rodent model [46]. Therefore, DNA methylation might be a target for pharmacological interventions for alcohol dependency [46]. In addition, DNA methylation could be a good biomarker of alcohol consumption [47]. Exposure to ethanol during adolescence might upregulate DNA methyltransferase activity, which can induce the hypermethylation of various genes such as those responsible for coding neuropeptide Y (NPY) and BDNF [48]. Furthermore, prenatal exposure to alcohol may generally trigger epigenetic modifications depending on the development stage, which may contain increased histone acetylation and/or reduced DNA/histone methylation [49]. In this regard, alcohol withdrawal may lead to dysregulated histone acetylation via the increased expression of histone deacetylase (HDAC) in some brain areas [50]. Hence, treatment with HDAC inhibitors can amend negative emotional conditions induced by alcohol withdrawal [50]. However, histone acetylation in the brain of patients exhibiting depression-like behavior during withdrawal after alcohol exposure may require further intensive examination.

## 5. Connection between Gut Microbiota and Alcohol Dependency

**Figure 2:**
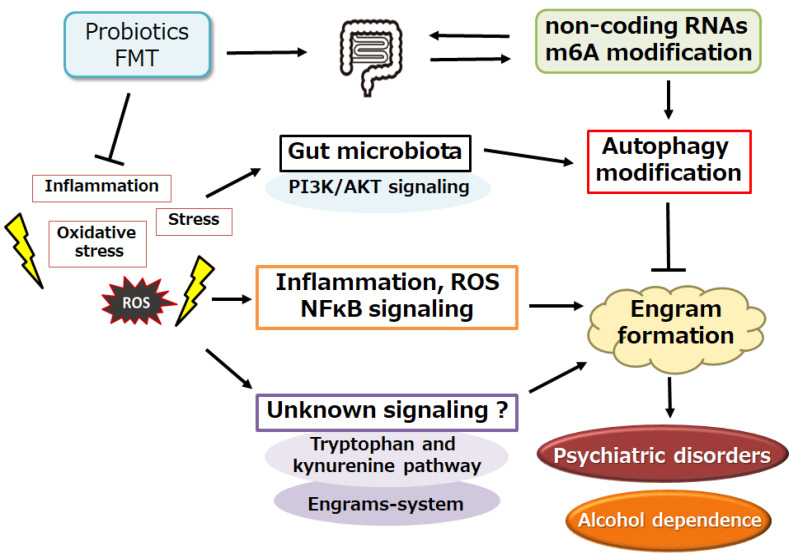
Schematic diagram of the possible strategies against the pathology of alcohol dependence. Several kinds of probiotics and/or fecal microbiota transplantation (FMT) may assist with the alteration of gut microbiota for the modification of autophagy, which may be advantageous for the inhibition of several engram formations, which may consequently improve the pathology of psychiatric disorders including alcohol dependence. Note that some of the significant activities such as autophagy initiation, inflammatory response, and ROS production have been omitted for clarity.

The gut microbiota has various effects on host physiology, including host metabolism, the development of the immune system, and even behaviors [51]. The complex interplay between the gut, stress, and eating/drinking behavior may facilitate new therapeutic targets for stress-related psychiatric disorders [51]. Remarkably, short-chain fatty acids (SCFAs), namely acetate, propionate, and butyrate, might be mediators of microbiota–gut–brain interactions on the stress response and/or eating/drinking behavior. In fact, various metabolites from the gut microbiome including SCFAs have been proved to regulate the histone acetylation process [52]. The microbiota–gut–brain axis is a bidirectional route of homeostatic communication via epigenetic mechanisms of diverse metabolites such as SCFAs. Thus, a modulation of the gut microbiota via diet or lifestyle can regulate neuron/brain inflammation via certain epigenetic mechanisms [53], which might be effective for enhancing emotional well-being and/or treating depressive disorders [54]. As important constituents of epigenetics by gut microbial metabolites and/or fermentation products, several miRNAs with epigenetic mechanisms have vital roles in various physiological homeostasis mechanisms [55,56]. For example, microbial acetate and/or butyrate might alleviate obesity with the regulation of host miRNAs [57]. In addition, there were close intricate interactions between gut microbiota, inflammation, and differential miRNAs, suggesting that ncRNAs may possess a potential role in the protection of the host against life-related diseases such as atherosclerosis [58]. Interestingly, it has been shown that circRNAs and the gut microbiome can interact to influence the growth of cancer cells [59]. Similarly, the expression of lncRNAs could be repressed by gut microbiota [60]. Increasing data may indicate that regulatory ncRNAs including miRNAs, circRNAs, and lncRNAs can influence the host–microta connection; these data show that regulatory ncRNAs may be potential biomarkers in microbiome-associated disorders including diabetes and cancers [61] (Figure 2).

In addition, the gut microbiota has an effect on host m6A mRNA modifications, which is another demonstration of the interaction between gut commensal bacteria and their hosts [62]. As well, the host m6A modification can also influence the gut microbiome by provoking gut inflammatory responses [63]. Possibly, *Lactobacillus plantarum* and/or *Akkermansia muciniphila* can influence the specific m6A modifications, which might emphasize epitranscriptomic modifications as a form of communication between gut commensal bacteria and the host [62,64]. The presence of a certain gut microbiome may also account for the significantly elevated m6A levels in the intestine [65]. Thus, m6A methylation is indeed involved in the host–gut microbiota crosstalk. On the one hand, substantial studies have suggested that the enteric microbiome is a key mediator of m6A modification. In general, a number of ncRNAs and/or m6A modification have been implicated in the onset and progression of drug addiction [66,67]. Therefore, the gut–brain axis may be the key to the homeostasis of the CNS, which may regulate several neuro-behaviors [68]. The gut microbiome could also affect drug bioavailability, blood–brain barrier (BBB) permeability, and social behaviors [69]. Developing microbiota-based interventions such as prebiotics, probiotics, FMT, or metabolite supplementation might be an exciting tactic for treating psychiatric disorders, probably including alcohol dependency.

## 6. A Possible Tactic with Alteration of Gut Microbiota against Alcohol Dependency

Too much alcohol consumption may induce gut dysbiosis, an imbalance in gut microbiota, through several mechanisms. Consequently, chronic alcohol exposure can reduce the production of mucus and several peptides, which may intrude the intestinal barrier [70]. In addition, alcohol consumption frequency could be a robust factor for the change of gut microbiota [71]. Although pharmacological treatments exist, their effectiveness depends on the appropriate faithfulness to the prescribed regimen. Therefore, most patients with alcohol dependency are left untreated, and there is a need for additional, more effective therapies. Identifying some biological markers that predict the susceptibility to developing extreme alcohol-consumption behaviors may lead to an enhancement of good clinical care. Interestingly, relationships between gut microbiota and the behavioral characteristics of alcohol dependency have been described [72]. A specific microbiota composition is linked to addiction behaviors in a realistic model of alcohol dependency [72]. Based on these findings, newfangled therapeutic regimens should embrace gut microbiome manipulation, which may lessen alcohol intake and/or drinking activities. Indeed, alcohol consumption produces both direct and indirect consequences for the gut microbiota via metabolism, neuronal response, and immune inflammatory cascades. In particular, chronic inflammatory conditions may lead to alterations in several inflammatory mediators that can activate the nuclear factor kappa B (NF-kB) signaling pathway, leading to neuronal damage/apoptosis in glial and/or neuronal cells [73]. It is important to note that not all patients with alcohol dependency have dysbiosis and/or increased gut epithelial disruption [74]. However, several effects of alcohol on the gut microbiome might contribute to increased alcohol consumption. Therefore, the use of probiotics, prebiotics, or FMT may deserve further investigation as therapeutic tactics for alcohol dependency [74]. At present, however, the application of FMT as a therapeutic approach is still in the investigatory stages [74]. Stool donor-to-recipient disease transfer is a great concern of FMT. Furthermore, the long-term effect of FMT on the gut microbiota and the brain still needs to be determined. [75] (Figure 2).

Interestingly, an antidepressant, arketamine (also termed R-ketamine), may restore the altered composition of gut microbiota of rodents with depression-like behaviors, indicating the beneficial effects of R-ketamine. Ketamine is a racemic mixture composed of two enantiomers, R-ketamine and esketamine (S-ketamine). Both enantiomers have exhibited antidepressant effects, whose effects are attributed to distinct pharmacological activities including the NMDA-channel and/or opioid receptor. It has been shown that antidepressant-like effects of both ketamines might be partly mediated by the alteration of gut microbiota [76]. Ketamine could potentially activate several biochemical signaling pathways, which may eventually lead to the inhibitory phosphorylation of the GSK3β molecule in microglia [77]. Remarkably, S-ketamine exerts neuroprotective effects via enhancing autophagy and lessening oxidative stress; the mechanism comprises AMPK/mTOR-dependent autophagy and/or the antioxidant system [78]. Amazingly, S-ketamine significantly altered the abundance of intestinal microbiota including *Adlercreutzia equolifaciens* and *Akkermansia muciniphila* [79]. It has been revealed that the regulation of NFAT signaling by miR-149 might play a role in the tenacious prophylactic effects of R-ketamine in inflammation, and that gut microbiota can regulate the gene expression of miRNAs via the gut–brain axis [80]. Increased miR-149 expression may be related to the reduced glial cell numbers in patients diagnosed with familial bipolar disorder [81]. Because miR-149 has been shown to inhibit glial proliferation, increased miR-149 expression is also consistent with the pathology of depressive disorders [82]. However, there are rarely reports showing the role of other miRNAs or m6A modification on the prophylactic effects of ketamine and its enantiomers in brain neuroinflammation disorders.

## 7. Future Perspectives

**Figure 3:**
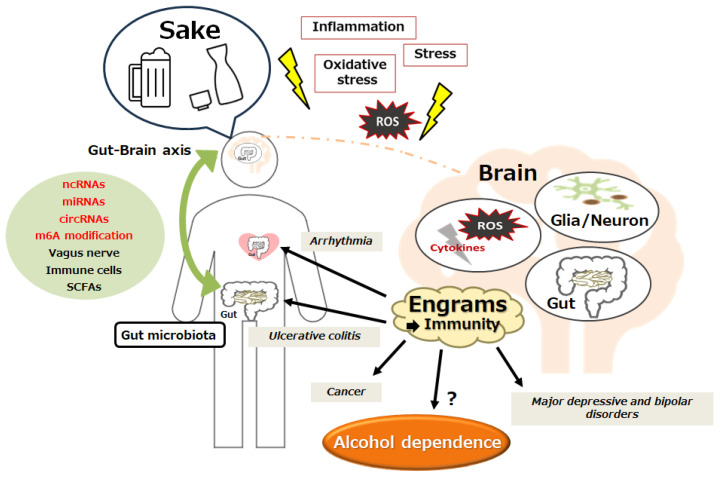
Schematic outline for the pathogenesis of immune-related disorders such as bipolar disorder, major depressive disorder, cardiovascular diseases, chronic kidney disease, acute kidney injury, inflammatory bowel disease, and alcohol dependence. The gut–brain axis, with the utilization of ncRNAs, m6A modification, and/or short chain fatty acids (SCFAs), may contribute to the pathogenesis of immune-related disorders via the construction of several “engrams” in the brain. Inflammation with reactive oxygen species (ROS) might be also involved in the pathway for the alteration of immune cells. Note that several significant activities such as anti-inflammatory reactions and/or cytokine induction have been absent for clarity. “?” means for author speculation.

It might be a great risk to use ketamine for improving alcohol dependence. In general, the use of both esketamine and racemic ketamine can lead to adverse events. In addition, it would be difficult to state whether ketamine abuse or alcohol abuse is more harmful. Based on the hypothesis that ketamine may improve alcohol dependency via the mechanism of improving autophagy in neuronal cells, dietary interventions would be possible for the treatment of alcohol dependency. This is because the modification of the gut microbiome is safely imaginable via dietary changes, which could also contribute to the alteration of ncRNA production and/or m6A modification in various cells [83,84]. In fact, some dietary supplements are acting through different mechanisms to reduce alcohol relapse [85]. As for prebiotics and/or probiotics, those interventions may be somewhat inadequate for treatment in regard to the improvement by autophagy [86,87]. Some additional factors and/or signaling activation might be required for improved dietary interventions, even against alcohol dependency. In addition to modulating the gut microbiome, for example, metformin could exert its advantageous effect by influencing mitochondrial function and/or renovating redox balance [88]. Moreover, several modules involved in the tryptophan and kynurenine pathway may also be plausible for the dietary intervention, which has been shown to be linked to various immune-related diseases including major depressive and/or bipolar disorders [89]. In short, formed by recurring inflammatory conditions, an “engram” might be devoted to a gentle development of these diseases [89]. In the brain, the engram memory system could retain the information of a certain inflammation in the body, which may be crucially involved in the pathogenesis of immune-related diseases, in which several immunity-linked processes might be associated with certain neuronal responses to memory engrams [90]. Therefore, forgetting or emptying the bad memory of “engrams” could be promising for the prevention and/or treatment against immune-related diseases, as well as cancer, cardiac arrhythmia, and/or neurodegenerative diseases [83,91,92] (Figure 3). If that is the case with alcohol dependence, a certain adjustment of the “engram” through the alteration of the gut microbiota might be helpful as a notable treatment tactic against alcohol dependence. Future work should precisely explain how this engram pathway could interfere with the progression of alcohol dependency at the molecular level (Figure 3).

## 8. Conclusions

Several ncRNAs and/or m6A modification may be involved in the instigation of alcohol dependency, and the relationship between the gut and brain can play an important role. In addition, the connection between the brain and immunity might also influence the development of alcohol dependency. An in-depth knowledge of the roles of ncRNAs and m6A in the gut microbiome may be valuable for the development of a novel treatment against alcohol dependence.

## Abbreviations

BBB	Blood–brain barrier
BDNF	brain-derived neurotrophic growth factor
CNS	central nervous system
circRNA	circular RNA
FMT	fecal microbiota transplantation
HDAC	histone deacetylase
lncRNA	long non-coding RNA
mRNA	messenger RNA
m6A	methylation of N6 adenosine
ncRNA	non-coding RNA
NF-kB	nuclear factor kappa B
NPY	neuropeptide Y
piRNAs	piwi interacting RNAs
siRNAs	small interfering RNAs
ROS	reactive oxygen species
SCFA	short-chain fatty acid
siRNA	short interference RNA
R-ketamine	arketamine
S-ketamine	esketamine
